# The Associations of Cerebrospinal Fluid ApoE and Biomarkers of Alzheimer’s Disease: Exploring Interactions With Sex

**DOI:** 10.3389/fnins.2021.633576

**Published:** 2021-03-03

**Authors:** Ying Liu, Jing-Hui Song, Wei Xu, Xiao-He Hou, Jie-Qiong Li, Jin-Tai Yu, Lan Tan, Song Chi, and Alzheimer’s Disease Neuroimaging Initiative

**Affiliations:** ^1^Department of Neurology, The Affiliated Hospital of Qingdao University, Qingdao, China; ^2^Department of Neurology, Qingdao Municipal Hospital, Qingdao University, Qingdao, China; ^3^Department of Neurology and Institute of Neurology, Huashan Hospital, Shanghai Medical College, Fudan University, Shanghai, China

**Keywords:** Alzheimer’s disease, cerebrospinal fluid, Apolipoprotein E, sex, amyloid, tau, ADNI

## Abstract

**Background:**

Sex-related difference in Alzheimer’s disease (AD) has been proposed, and apolipoprotein E (ApoE) isoforms have been suggested to be involved in the pathogenesis of AD.

**Objective:**

We aimed to explore whether cerebrospinal fluid (CSF) ApoE is associated with AD biomarkers and whether the associations are different (between sexes).

**Methods:**

Data of 309 participants [92 with normal cognition, 148 with mild cognitive impairment (MCI), and 69 with AD dementia] from the Alzheimer’s Disease Neuroimaging Initiative (ADNI) were cross-sectionally evaluated with the multiple linear regression model and longitudinally with the multivariate linear mixed-effects model for the associations of CSF ApoE with AD biomarkers. Sex–ApoE interaction was used to estimate whether sex moderates the associations of CSF ApoE and AD biomarkers.

**Results:**

Significant interactions between CSF ApoE and sex on AD biomarkers were observed [amyloid-β (Aβ): *p* = 0.0169 and phosphorylated-tau (p-tau): *p* = 0.0453]. In women, baseline CSF ApoE levels were significantly associated with baseline Aβ (*p* = 0.0135) and total-tau (t-tau) (*p* < 0.0001) as well as longitudinal changes of the biomarkers (Aβ: *p* = 0.0104; t-tau: *p* = 0.0110). In men, baseline CSF ApoE levels were only correlated with baseline p-tau (*p* < 0.0001) and t-tau (*p* < 0.0001) and did not aggravate AD biomarkers longitudinally.

**Conclusion:**

The associations between CSF ApoE and AD biomarkers were sex-specific. Elevated CSF ApoE was associated with longitudinal changes of AD biomarkers in women, which indicates that CSF ApoE might be involved in the pathogenesis of AD pathology in a sex-specific way.

## Introduction

Alzheimer’s disease (AD) is the leading cause of dementia characterized by abnormal accumulation of β-amyloid (Aβ) (Hardy and Selkoe, 2002) and aggregation of hyperphosphorylated tau in the brain (Zempel and Mandelkow, 2014). A greater number of women have been diagnosed with AD compared with men according to the epidemiological indicators (Alzheimer’s Association, 2014), which can be partially explained by the sex-related differences in neural anatomy and function (Ingalhalikar et al., 2014; Ritchie et al., 2018). Addressing the sex-specific variation is crucial for the development of precise and effective therapeutics in AD.

It is well recognized that Apolipoprotein E (*APOE*) genotype is the strongest genetic risk factor for late-onset AD. Recent studies have found that the effect of *APOE* gene on AD is modified by sex (Altmann et al., 2014; Wang et al., 2019). Women between the ages of 65 and 75 with *APOE* ε3/ε4 have an increased risk of developing mild cognitive impairment (MCI) or AD compared with men (Neu et al., 2017), and the association between *APOE* ε*4* and cerebrospinal fluid (CSF) tau level is stronger among women than men (Hohman et al., 2018). Animal studies also highlighted that impaired cognition and decreased presynaptic density were only observed in female *APOE* ε*4* knockout mice (Rijpma et al., 2013; Pontifex et al., 2018). On the other hand, *APOE* ε*2* was implicated to play a protective role in either men (Altmann et al., 2014; Zhao et al., 2015) or women (McFall et al., 2019).

Apolipoprotein E (ApoE), the *APOE* gene-encoded protein, has been suggested to be involved in a variety of pathogenic processes of AD. Several studies have reported that ApoE acts on Aβ deposition (Strittmatter et al., 1993; Bales et al., 1999; Tokuda et al., 2000; Baker-Nigh et al., 2016) and disrupts its clearance (Deane et al., 2008) in an isoform-dependent way. The lipidation status of ApoE has also been suggested to influence degradation of soluble Aβ peptides (Jiang et al., 2008). ApoE not only binds to soluble Aβ but also competes for its clearance pathways in the brain (Verghese et al., 2013). Similarly, ApoE was found to accelerate tau spreading (Wadhwani et al., 2019) and positively correlate with tau protein level (Lindh et al., 1997; van Harten et al., 2017). Moreover, ApoE was proposed to play a role in neurotoxicity (Mahley and Huang, 2012), mitochondrial dysfunction (Chen et al., 2011), and blood–brain barrier permeability (Teng et al., 2017; Main et al., 2018), which are all key mechanisms to AD pathogenesis. Clinical findings showed that high CSF ApoE concentration could predict the clinical progression of *APOE* ε*4* carriers (van Harten et al., 2017), although no consensus has been reached for the association of CSF ApoE concentration with AD pathogenesis. Additionally, a recent study found that CSF ApoE mediated the positive association of *APOE* ε*4* with tau without affecting the inverse relation between *APOE* ε*4* and Aβ (Slot et al., 2019), indicating that CSF ApoE might be involved in AD pathology with mechanisms independent of those of *APOE* gene on AD pathology. However, whether the process is modified by sex has been mostly unexplored.

Interestingly, ApoE seems to be independently synthesized in the central nervous system and in the peripheral nervous system. It was found that most CSF ApoE was synthesized in the central nervous system, as it did not change to the donor’s phenotype after liver transplantation (Linton et al., 1991). Moreover, a study in mice revealed that plasma ApoE could not cross the blood–CSF barrier (Liu et al., 2012). It is known that the composition of CSF is similar to that of extracellular fluid of the brain tissue, and the CSF biomarkers are valid proxies for neuropathologic changes of AD (Jack et al., 2018). Therefore, we aim to explore whether there are sex-related associations of CSF ApoE and AD biomarkers using Alzheimer’s Disease Neuroimaging Initiative (ADNI) database.

## Materials and Methods

### Study Design and Data Sources

The study was designed to investigate whether there are sex-specific associations of CSF ApoE with well-validated AD biomarkers by means of cross-sectional and longitudinal analyses. Data were downloaded from the ADNI database^1^, which was launched in 2003 as a public–private partnership, led by principal investigator Michael W. Weiner, MD. It longitudinally collected detailed clinical, imaging, and laboratory data from more than 50 sites across the United States and Canada (the most recent information on the ADNI is available at http://www.adni-info.org). The filenames that contained the ApoE level are Biomarkers Consortium CSF Proteomics Project RBM Multiplex Data and Primer (Zip file) and Biomarkers Consortium Plasma Proteomics Project RBM Multiplex Data and Primer (Zip file) in the website https://ida.loni.usc.edu/pages/access/studyData.jsp?categoryId=11&subCategoryId=33. Institutional review boards of all participating institutions approved the ADNI, and written informed consent was obtained from all participants or authorized representatives. This study was approved by the ethics committee of our institution (IRB number: QYFYWZLL26124).

### Participants

Our cohort consists of all cognitively normal (CN) controls and MCI and AD participants from ADNI-1. Detailed inclusion and exclusion criteria have been reported previously (Petersen et al., 2010). The inclusion criteria of this study are as follows: (1) available baseline CSF ApoE and AD biomarkers (Aβ, t-tau, and p-tau) measurements; (2) sufficient data of sociodemographic characteristics (age, sex, and education) and clinical evaluations [*APOE* ε4 genotype, baseline cognitive diagnosis, body mass index (BMI), history of cardiovascular disease, dyslipidemia, hypertension, and depression]. After excluding one participant without BMI information, 309 participants with sufficient data of baseline CSF ApoE and other information were included in the study. Besides, we included 356 participants with baseline plasma ApoE and sufficient data of sociodemographic and clinical information to evaluate the associations of baseline plasma ApoE with AD biomarkers.

### Exposure Measures

The exposure measures include CSF ApoE and plasma ApoE. CSF ApoE was measured by the multiplex Human Discovery MAP^TM^ panel on a Luminex 100 platform. Plasma was collected after an overnight fasting and plasma ApoE was measured by a 190-analyte multiplex immunoassay panel on the Luminex xMAP platform (see papers on methods and procedures available in http://www.adni-info.org).

### Outcome Measures

Outcome measures include CSF Aβ, t-tau, and p-tau both at baseline and follow-ups. CSF was sampled through lumbar puncture and CSF Aβ, t-tau, and p-tau were measured by a multiplex xMAP platform with the INNOBIA AlzBio3 kit (Innogenetics, Ghent, Belgium) (Olsson et al., 2005). Participants have at least one follow-up measurement and the longest follow-up period is 5 years. A total of 870 measurements were included in the study. Longitudinal CSF data have been analyzed and described previously in detail (Toledo et al., 2013).

### Covariates

Demographic information, *APOE* ε4 genotype, baseline cognitive diagnosis, and medical history were downloaded from the ADNI database. Other risk factors that might potentially affect the progress of AD were included in the present study, such as history of cardiovascular disease (i.e., myocardial infarction, intermittent claudication, angina, heart failure, and other evidence of coronary disease), dyslipidemia (i.e., hypercholesterolemia, low levels of high-density lipoprotein cholesterol, and hypertriglyceridemia), hypertension, BMI, and depression. Selection of the covariates was based on the previous studies (Livingston et al., 2017; Ferretti et al., 2018).

### Statistical Analyses

Clinical and demographic variables of different groups were compared using the Kruskal–Wallis test for non-parametric variables and the Chi-square test for categorical variables. Spearman rank correlation was used for correlations between CSF ApoE and AD biomarkers.

Multiple linear regression model was performed to explore the cross-sectional associations of baseline CSF ApoE and AD biomarkers, with all outcome variables being standardized to *z* scores before entering into the model. Two predefined models were used with the following covariates (model 1: age, sex, *APOE* ε4 carrier status, education, and baseline cognitive diagnosis; model 2: model 1 plus cardiovascular disease, hypertension, BMI, dyslipidemia, and depression). Interaction between CSF ApoE and sex was further conducted in model 2 to evaluate the sex-specific associations of baseline CSF ApoE with AD biomarkers.

Multivariate linear mixed-effects model with random intercepts and slopes (time), termed time-by-ApoE interaction, was used to determine the associations of baseline CSF ApoE and longitudinal changes of AD biomarkers adjusted for the covariates in model 2. All outcome variables in the model were standardized to *z* scores to facilitate comparisons between modalities. Further analysis with a time-by-ApoE-by-sex interaction was included in the longitudinal analyses to evaluate whether CSF ApoE interacted with sex in association with longitudinal changes of AD biomarkers over the follow-up periods. All lower-order interactions of this three-way interaction term were included in the model.

A two-tailed *p* < 0.05 was considered statistically significant, except for the interaction analyses in the cross-sectional studies (*p* < 0.1), which aims to explore whether there was any potential interaction. R software, version 3.4.4 (R-project.org/), was used for all statistical analyses.

## Results

### Sample Characteristics

Baseline characteristics of the 309 participants are shown in Table 1. In brief, 92 CN controls (mean age, 75.70 ± 5.45; male, 50.0%), 148 MCI patients (mean age, 74.84 ± 7.23; male, 68.9%), and 69 AD patients (mean age, 74.94 ± 7.61; male, 56.5%) with available baseline CSF ApoE from ADNI-1 cohort were included. The level of CSF ApoE in men is higher than that in women (Table 1), and significant difference of CSF ApoE level among different *APOE* ε4 carrier status was found (Table 2). No significant differences of CSF ApoE were observed among the three study groups in the univariate analysis. There was also no significant difference in sex-stratified AD biomarkers (Table 1).

**TABLE 1:** Demographic and clinical characteristics.

	Women				Men				CN *N* = 92	MCI *N* = 148	AD *N* = 69	*P*_1_	*P*_2_
	CN *N* = 46	MCI *N* = 46	AD *N* = 30	Total *N* = 122	CN *N* = 46	MCI *N* = 102	AD *N* = 39	Total *N* = 187					
Age, years	76.26 ± 5.15	72.18 ± 6.78	73.76 ± 7.72	74.11 ± 7.54	75.15 ± 5.73	76.40 ± 7.14	75.85 ± 7.50	75.78 ± 6.88	75.70 ± 5.45	74.84 ± 7.23	74.94 ± 7.61	0.0309	0.8281
*APOE*ε4 carriers	7 (17.39)	29 (63.04)^*a*^	22 (73.33)^*a*^	58 (47.54)	15 (34.78)	50 (49.02)	27 (69.23)	92 (49.20)	22 (23.91)	79 (53.38)	49 (71.01)	0.8662	<0.0001
BMI, kg/m^2^	26.26 ± 5.17	25.16 ± 4.26	24.15 ± 3.64	25.33 ± 4.21	26.74 ± 4.28	25.79 ± 3.59	26.03 ± 3.45	26.07 ± 3.74	26.50 ± 4.73	25.60 ± 3.80	25.21 ± 3.63	0.0652	0.1877
Educational level, years	14.74 ± 2.69	15.24 ± 2.88	14.3 ± 2.64	14.82 ± 2.80	16.52 ± 2.95	16.29 ± 2.92	15.82 ± 3.09	16.25 ± 2.96	15.63 ± 2.95	15.97 ± 2.94	15.16 ± 2.98	<0.0001	0.3382
CSF ApoE	7.20 ± 2.24	6.42 ± 1.74	5.96 ± 2.11	6.601 ± 48.33	7.41 ± 2.17	7.56 ± 2.40	6.91 (2.39)	7.39 ± 2.35	7.30 ± 2.20	7.21 ± 2.27	6.50 ± 2.31	0.0046	0.0571
CSF Aβ	212.80 ± 51.44	156.10 ± 44.37	141.50 ± 30.94	173.90 ± 59.42	203.20 ± 55.40	162.10 ± 51.37	141.40 ± 38.16	167.90 ± 54.28	160.32 ± 53.38	160.24 ± 49.23^*a*^	141.41 ± 34.96^*a,b*^	0.3074	<0.0001
CSF t-tau	69.93 ± 27.44	115.86 ± 52.23	137.8 ± 64.56	103.94 ± 64.05	67.76 ± 25.52	110.59 ± 52.79	110.25 ± 52.07	94.53 ± 49.74	68.85 ± 26.37	105.33 ± 52.92^*a*^	122.24 ± 59.01^*a,b*^	0.1197	<0.0001
CSF p-tau	23.44 ± 10.35	38.30 ± 15.43	42.38 ± 16.08	33.70 ± 19.27	26.13 ± 15.93	35.16 ± 15.51	40.13 ± 23.70	33.97 ± 18.19	24.79 ± 13.43	36.14 ± 15.50^*a*^	41.11 ± 20.63^*a,b*^	0.7774	<0.0001
Cardiovascular disease	8 (17.39)	11 (23.91)	5 (16.67)	24 (19.67)	23 (50.00)	33 (32.35)	19 (48.72)	75 (40.11)	31 (33.70)	44 (29.73)	24 (34.78)	0.0003	0.6987
Hypertension	25 (54.35)	18 (39.13)	16 (53.33)	59 (48.36)	20 (43.48)	53 (51.96)	18 (46.15)	91 (48.66)	45 (48.91)	71 (47.97)	34 (49.28)	1.0000	0.9806
Hyperlipoidemia	17 (36.96)	19 (41.30)	15 (50.00)	51 (41.80)	23 (50.00)	48 (47.06)	20 (51.28)	91 (48.66)	40 (43.48)	67 (45.27)	35 (50.72)	0.2864	0.6417
Depression	5 (10.87)	14 (30.43)	14 (46.67)	33 (27.05)	3 (6.52)	16 (15.69)	8 (20.51)	27 (14.44)	8 (8.70)	30 (2.02)	22 (31.88)	0.0095	0.0011
Mean follow-up, y	2.61 ± 1.69	1.98 ± 1.39	1.57 ± 0.86	2.16 ± 1.46	2.63 ± 1.57	2.31 ± 1.47	1.18 ± 0.51	2.11 ± 1.45	2.62 ± 1.62	2.21 ± 1.45	1.35 ± 0.70^*a,b*^	0.6999	<0.0001

*BMI, body mass index; CSF, cerebrospinal fluid; ApoE, Apolipoprotein E; Aβ, amyloid-β; p-tau, phosphorylated tau; t-tau, total tau; MCI, mild cognitive impairment; CN, cognitively normal; AD, Alzheimer’s disease. Categorical data are shown as *n* (%) and continuous data are shown as mean ± SD. ***P*_1_** value is comparison of the total participants in female and male groups. ***P*_2_** value is comparison of the total participants in CN, MCI, and AD groups. ^*a*^*P* < 0.01 compared to the total participants in CN. ^*b*^*P* < 0.01 compared to the total participants in MCI.*

**TABLE 2:** Demographic and clinical characteristics of participants stratified by *APOE* ε4 carrier status.

	*APOE* ε4 non-carriers *N* = 159	With one *APOE* ε4 allele *N* = 114	The *APOE* ε4/ε4 genotype *N* = 36	*P*
Age, years	75.76 ± 6.90	75.33 ± 6.45	71.61 ± 6.85	0.0040
Men	95 (59.75)	72 (63.16)	20 (55.56)	0.6897
BMI, kg/m^2^	26.31 ± 4.50	25.07 ± 3.42	25.69 ± 3.82	0.1102
Educational level, years	15.90 ± 3.01	15.61 ± 3.07	15.00 ± 2.20	0.1203
CSF ApoE	7.46 ± 2.29	6.76 ± 2.23	6.40 ± 2.08	<0.0001
CSF Aβ	199.00 ± 53.84	146.70 ± 34.26	117.90 ± 22.52	<0.0001
CSF t-tau	82.96 ± 48.17	112.50 ± 51.85	120.62 ± 50.64	<0.0001
CSF p-tau	28.24 ± 15.57	38.92 ± 15.98	42.74 ± 20.49	<0.0001
Cardiovascular disease	54 (33.96)	36 (31.58)	9 (25.00)	0.5768
Hypertension	85 (53.46)	49 (42.98)	16 (44.44)	0.2027
Hyperlipoidemia	62 (38.99)	57 (50.00)	23 (63.89)	0.0142
Depression	29 (18.24)	24 (21.05)	7 (19.45)	0.8454
Mean follow-up, years	2.31 ± 1.53	2.01 ± 1.34	1.81 ± 1.37	0.0624

*BMI, body mass index; CSF, cerebrospinal fluid; ApoE, Apolipoprotein E; Aβ, amyloid-β; p-tau, phosphorylated tau; t-tau, total tau; MCI, mild cognitive impairment; CN, cognitively normal; AD, Alzheimer’s disease. Categorical data are shown as *n* (%) and continuous data are shown as mean ± SD.*

During the 5-year-follow-up, 2 CN controls and 85 MCI patients progressed to AD, and the mean CSF ApoE is 7.03 μg/ml when these two groups are combined as a whole. The mean CSF ApoE is 7.37 μg/ml in study participants who did not progress to AD. No significant differences of CSF ApoE were observed between those who progressed to AD and those who did not (*p* = 0.3709).

### Associations of CSF ApoE With CSF Biomarkers at Baseline

**FIGURE 1:**
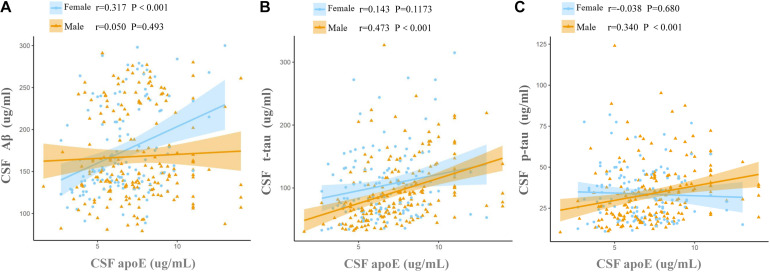
Associations of baseline CSF ApoE concentration with AD biomarkers. Fit lines are shown for individual sex groups. **(A)** Association of CSF ApoE and amyloid-β (Aβ); **(B)** association of CSF ApoE and total tau (t-tau); **(C)** association of CSF ApoE and phosphorylated tau (p-tau). The coefficients *r* and *P* values are for Spearman rank correlation in each sex cohort.

Cerebrospinal fluid ApoE levels were positively associated with t-tau [β (s.e.): 0.169 (0.021), *p* < 0.0001] and p-tau [β (s.e.): 0.098 (0.023), *p* < 0.0001], but not with Aβ [β (s.e.): 0.014 (0.020), *p* = 0.4742] for all the participants at baseline. Significant CSF ApoE-by-sex interactions with Aβ [β (s.e.): −0.101 (0.042), *p* = 0.0169] and p-tau [β (s.e.): 0.098 (0.049), *p* = 0.0453] were observed (Table 3). Further analysis stratified by sex showed that both women and men exhibited significant associations of CSF ApoE with t-tau, while the association of CSF ApoE with Aβ existed only in women, and the association of CSF ApoE with p-tau existed only in men (Table 4). The results were in line with those in Spearman’s correlation (Figure 1), which showed that CSF ApoE level was only positively associated with Aβ (*r* = 0.317, *p* < 0.001) in women, whereas the positive associations with t-tau (*r* = 0.473, *p* < 0.001) and p-tau (*r* = 0.340, *p* < 0.001) were only found in men.

**TABLE 3:** Associations of CSF ApoE with CSF biomarkers at baseline.

	Model 1		Model 2			
	CSF ApoE		CSF ApoE		CSF ApoE × sex	
	β (s.e.)	*P*	β (s.e.)	*P*	β (s.e.)	*P*
CSF Aβ	0.001 (0.002)	0.5400	0.014 (0.020)	0.4742	−0.101 (0.042)	0.0169*
CSF t-tau	0.176 (0.021)	<0.0001*	0.169 (0.021)	<0.0001*	0.029 (0.045)	0.5216
CSF p-tau	0.102 (0.023)	<0.0001*	0.098 (0.023)	<0.0001*	0.098 (0.049)	**0.0453**

Data are presented as standardized regression coefficients β and standard error (s.e.) with *P* values. *Signifies effect is significant when correcting for multiple comparisons (Bonferroni). Bold values mean statistically significant.

**TABLE 4:** Stratified analysis of baseline data.

	CSF Aβ		CSF t-tau		CSF p-tau	
	β (s.e.)	*P*	β (s.e.)	*P*	β (s.e.)	*P*
Female	0.093 (0.037)	0.0135*	0.170 (0.037)	<0.0001*	0.036 (0.039)	0.3576
Male	-0.023 (0.024)	0.3509	0.181 (0.027)	<0.0001*	0.122 (0.029)	<0.0001*

Data are presented as standardized regression coefficients β and standard error (s.e.) with *P* values. The regression analysis was analyzed in model 2. *Signifies effect is significant when correcting for multiple comparisons (Bonferroni).

### Associations of Baseline CSF ApoE With Longitudinal Changes of CSF Biomarkers

**FIGURE 2:**
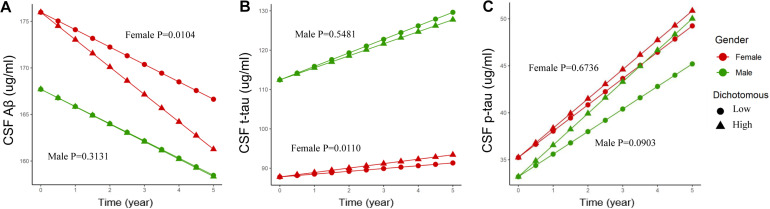
Associations of different CSF ApoE concentrations and longitudinal changes of CSF biomarkers. Longitudinal CSF Aβ **(A)**, CSF t-tau **(B)**, and CSF p-tau **(C)** change based on dichotomy of CSF ApoE level. For better visual display, the baseline values were held at the adjusted means for each sex group. High: ≥7.0 μg/ml, Low: <7.0 μg/ml. 7.0 μg/ml is the median for the baseline CSF ApoE. *P* value was the result of interaction between time and CSF ApoE level for linear mixed-effects models in each sex cohort.

Baseline CSF ApoE was not associated with longitudinal changes of CSF Aβ [β (s.e.): −0.002 (0.004), *p* = 0.6879], t-tau [β (s.e.): 0.005 (0.005), *p* = 0.2823], or p-tau [β (s.e.): 0.012 (0.009), *p* = 0.1796] in the multivariate linear mixed-effect model in all the participants (Table 5). However, a three-way interaction of ApoE-by-sex-by-time was found to be significantly associated with changes of Aβ [β (s.e.): 0.023 (0.009), *p* = 0.0096] and t-tau [β (s.e.): −0.027 (0.010), *p* = 0.0088]. Further analysis stratified by sex showed that ApoE was negatively associated with the longitudinal change of Aβ and positively associated with the change of t-tau in women (Table 6). No significant associations were found between baseline CSF ApoE and longitudinal changes of AD biomarkers in men. In women, baseline CSF ApoE was correlated with longitudinal changes of Aβ [β (s.e.): −0.018 (0.007), *p* = 0.0104] and t-tau [β (s.e.): 0.020 (0.007), *p* = 0.0110] during the following 5 years. As seen in Figure 2, participants with high CSF ApoE levels had a faster decrease of Aβ and increase of t-tau compared with those with low ApoE level during the following 5 years.

**TABLE 5:** Associations of baseline CSF ApoE with longitudinal changes of CSF biomarkers.

	Model 1		Model 2			
	CSF ApoE		CSF ApoE × time		CSF ApoE × sex × time	
	β (s.e.)	*P*	β (s.e.)	*P*	β (s.e.)	*P*
CSF Aβ	−0.002 (0.004)	0.6880	−0.002 (0.004)	0.6879	0.023 (0.009)	0.0096*
CSF t-tau	0.005 (0.005)	0.2838	0.005 (0.005)	0.2823	−0.027 (0.010)	0.0088*
CSF p-tau	0.012 (0.009)	0.1780	0.012 (0.009)	0.1796	0.013 (0.019)	0.5060

Data are presented as standardized regression coefficients β and standard error (s.e.) with *P* values. *Signifies effect is significant when correcting for multiple comparisons (Bonferroni).

**TABLE 6:** Stratified analysis of longitudinal data.

	CSF Aβ		CSF t-tau		CSF p-tau	
	β (s.e.)	*P*	β (s.e.)	*P*	β (s.e.)	*P*
Female	−0.018 (0.007)	0.0104*	0.020 (0.007)	0.0110*	0.007 (0.016)	0.6736
Male	0.006 (0.005)	0.3131	−0.004 (0.007)	0.5481	0.020 (0.012)	0.0903

Data are presented as standardized regression coefficients β and standard error (s.e.) with *P* values. The regression analysis was analyzed in model 2. *Signifies effect is significant when correcting for multiple comparisons (Bonferroni).

### Associations of CSF ApoE With CSF Sex Hormone-Binding Globulin at Baseline

**FIGURE 3:**
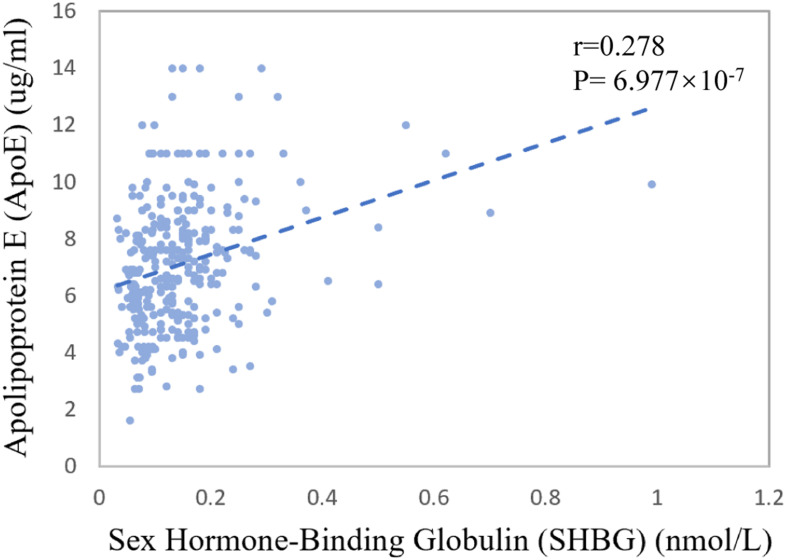
Association between CSF ApoE concentration and sex hormone-binding globulin at baseline. The coefficients *r* and *P* values are for Spearman rank correlation in the whole cohort.

The mean of CSF sex hormone-binding globulin (SHBG) was 0.14 nmol/L for CN control, 0.15 nmol/L for MCI patients, and 0.14 nmol/L for AD patients (*p* = 0.341). The Spearman correlation coefficient between baseline CSF SHBG and CSF ApoE was 0.278 (*p* = 6.977 × 10^–7^, Figure 3).

### Associations of Baseline Plasma ApoE With CSF AD Biomarkers

At baseline, a total of 263 participants have both plasma ApoE and CSF ApoE measurements, the levels of which showed a mild correlation (*r* = 0.17, *p* = 0.005, Supplementary Figure 1) in the analysis. Besides, plasma ApoE levels of the 356 participants were positively associated with t-tau [β (s.e.): 0.003 (0.001), *p* = 0.0462] and had a significant interaction with sex on t-tau [β (s.e.): 0.006 (0.003), *p* = 0.0659] at baseline. Further analysis stratified by sex showed that the association of plasma ApoE with t-tau existed only in men [women: β (s.e.): 0.003 (0.002), *p* = 0.1426; men: β (s.e.): 0.007 (0.003), *p* = 0.0177]. However, baseline plasma ApoE was not associated with longitudinal changes of Aβ, t-tau, or p-tau in all participants. Similarly, no significant three-way interaction of time × plasma ApoE × sex was found (Supplementary Table 1).

## Discussion

In this study, we observed significant sex-specific associations of CSF ApoE with AD biomarkers. In women, baseline CSF ApoE was significantly associated with both baseline CSF Aβ and t-tau as well as longitudinal changes of the biomarkers. However, the longitudinal associations were not observed in men, indicating that CSF ApoE could be considered as an early marker for AD in women.

Women have been found to have a higher risk of developing AD even after the prolonged life expectancy has been controlled (Li and Singh, 2014). Sex is a crucial variable in AD heterogeneity (Ferretti et al., 2018). Compared with men, women have a higher lifetime risk of developing AD (Seshadri et al., 1997) and are more likely to progress into severe clinical manifestations (Koran et al., 2017), have more extensive brain AD pathology (Corder et al., 2004; Barnes et al., 2005), and have faster brain atrophic rate measured by magnetic resonance imaging (Hua et al., 2010). Our results complement another mechanism of sex difference between CSF ApoE and major pathologies of AD, which might partly explain the greater disease burden of AD in women.

It is known that ApoE synergistically increases Aβ production (Sawmiller et al., 2019) and its deposit in the brain (Bales et al., 1999) and disrupts Aβ clearance process (Deane et al., 2008; Jiang et al., 2008; Verghese et al., 2013). In our current study, CSF ApoE was associated with increased baseline CSF Aβ and predicted its decline in the following 5 years in women. The result indicated that CSF ApoE might aggravate Aβ deposition in the brain (Bales et al., 1999) by acting as “pathological molecular chaperones” (Wisniewski and Frangione, 1992) to promote insolubility or neurotoxicity of CSF Aβ in the early stage of AD pathology (Hudry et al., 2019) and (or) by disrupting Aβ clearance process by competing for the same clearance pathways of soluble Aβ (Verghese et al., 2013). The level of CSF Aβ was abnormally low after the formation of insoluble amyloid plaque. In our findings, baseline CSF ApoE in women was associated with the longitudinal increase of t-tau without any association with p-tau. It is generally recognized that CSF t-tau is a biomarker for the intensity of neurodegeneration (Blennow and Zetterberg, 2018), and Aβ is the upstream of tau in the pathogenesis of AD by triggering tau from the normal state to toxic state (Bloom, 2014; Buckley et al., 2019). Therefore, besides the accumulation of upstream Aβ, CSF ApoE might promote neurodegeneration of AD via other mechanisms such as mitochondrial dysfunction (Yin et al., 2019), cytoskeletal alterations (Mahley and Huang, 2012), and inflammation (Shi and Holtzman, 2018). Our findings suggest that CSF ApoE can be an important promoter of the pathological process of AD in women.

In men, baseline CSF ApoE was only correlated with baseline tau pathology and was not associated with longitudinal AD biomarkers. A previous study has found that ApoE might affect tau pathogenesis independent of Aβ pathology (Shi et al., 2017). CSF ApoE was observed to increase after nerve injury (Aoki et al., 2003; Xu et al., 2006). These findings could partially explain the positive association of CSF ApoE with t-tau at baseline in both women and men. The results that CSF ApoE was not associated with the pathological deterioration of AD in men are in line with the previous findings using ADNI database in all participants (Toledo et al., 2014). However, after stratified by sex, we found that the association of ApoE and AD biomarkers was sex-specific and baseline CSF ApoE was only associated with the longitudinal changes of CSF Aβ and CSF t-tau in women.

We further explored the potential mechanism for the sex-specific association between CSF ApoE and AD neuropathology. Interestingly, we found that CSF SHBG, a major transport protein that modulates biologically active testosterone and estradiol, had a weak but significant positive correlation with CSF ApoE at baseline (*r* = 0.278, *p* = 6.977 × 10^–7^, Figure 3). Patients with AD were reported to have higher plasma SHBG levels, which may inactivate the functional testosterone and estradiol that are biologically neuroprotective (Xu et al., 2016). Prior work also demonstrated that the expression of *APOE* gene could be modulated by estrogen (Stone et al., 1997; Srivastava et al., 2008) and its receptors (Wang et al., 2006). Hence, we speculate that the sex-specific associations of CSF ApoE with AD biomarkers may be partially modulated by female sex hormones, which needs further exploration in the future. At the same time, we cannot ignore other potential mechanisms, such as that the half-life or production of ApoE in CSF is sex-specific.

The turnover rate of ApoE isoforms differs substantially in the central nervous system and in the peripheral nervous system (Wildsmith et al., 2012). In our study, we found a weak correlation between CSF ApoE and plasma ApoE. At baseline, plasma ApoE levels were positively associated with t-tau and had a significant interaction with sex on t-tau. However, baseline plasma ApoE was not associated with longitudinal changes of AD biomarkers, and there was no significant interaction of time × ApoE × sex. Hence, unlike CSF ApoE, plasma ApoE is not an early biomarker for AD cascade irrespective of sex. The result is in line with the findings in animal studies, which showed that ApoE in peripheral nervous system might function differently from ApoE in the central nervous system (Lane-Donovan et al., 2016), probably due to the fact that they cannot permeate the blood–brain barrier (Liu et al., 2012).

There are some limitations in this study. First, the small sample size and the potential sampling bias of ADNI (high proportion of men with AD) might limit the generalizability of these findings. Second, the missing longitudinal data might bias estimates of the longitudinal associations between baseline ApoE and AD biomarkers. Third, CSF ApoE with different ApoE isoforms (Yu et al., 2014), diverse origins (Brecht et al., 2004), and lipidation state (Jiang et al., 2008; Chernick et al., 2018) may have distinct function on neurodegeneration. In our study, we are unable to differentiate CSF ApoE isoforms, cellular origin, and lipidation state, all of which might be the potential factors affecting the association between CSF ApoE and AD biomarkers. Finally, although we have ruled out the confounding factors that could potentially affect AD progression, there are still possibilities that the covariates have causal relationships with CSF ApoE, which might reduce statistical power.

In summary, we found significant sex differences in the associations between CSF ApoE protein and AD biomarkers. In women, elevated CSF ApoE was associated with longitudinal changes of AD biomarkers, which might be an important promoter for the neurodegeneration of AD pathology. More work is needed to explore the potential mechanism underlying the role of ApoE in the pathogenesis of AD in women and how the association between sex hormones and ApoE influences AD pathology.

## Data Availability Statement

The original contributions presented in the study are included in the article/Supplementary Material, further inquiries can be directed to the corresponding author/s.

## Ethics Statement

The study was approved by the Medical ethics committee of Affiliated Hospital of Qingdao University (IRB number: QYFYWZLL26124).

## Author Contributions

J-TY, LT, and SC conceived this study, interpreted the data, and revised the manuscript. YL and WX determined the eligibility of the included studies and extracted the data independently. YL, J-HS, and X-HH conducted statistical analysis of the data and prepared all the figures. YL, J-QL, and X-HH drafted and modified the manuscript.

## Conflict of Interest

The authors declare that the research was conducted in the absence of any commercial or financial relationships that could be construed as a potential conflict of interest.

## Footnotes

1 http://adni.loni.usc.edu
