# Supplementary material for: The Associations of Cerebrospinal Fluid ApoE and Biomarkers of Alzheimer’s Disease: Exploring Interactions With Sex
Source: Front Neurosci. 2021 Mar 3;15:633576. doi: 10.3389/fnins.2021.633576 (PMC7968417; doi:10.3389/fnins.2021.633576)
Supplement: Supplementary file 1 [file Table_1.DOCX]

Supplementary Material

**Tables 1. Associations of baseline plasma ApoE levels with biomarkers of AD.**

|  | Model1 | |  | Model2 | | | | |
| --- | --- | --- | --- | --- | --- | --- | --- | --- |
|  | Plasma ApoE | |  | Plasma ApoE(×time) | |  | Plasma ApoE×sex(×time) | |
|  | β(s.e.) | P |  | β(s.e.) | P |  | β(s.e.) | P |
| Cross-sectional outcomes | | | | | | | | |
| CSF Aβ | 0.001(0.001) | 0.3985 |  | -0.001(0.001) | 0.3380 |  | 0.002(0.003) | 0.4981 |
| CSF t-tau | 0.003(0.002) | 0.0666 |  | 0.003(0.001) | **0.0462** |  | 0.006(0.003) | **0.0659** |
| CSF p-tau | 0.002(0.002) | 0.1078 |  | 0.003(0.002) | 0.0924 |  | 0.004(0.003) | 0.2346 |
| Longitudinal outcomes | | | | | | | | |
| CSF Aβ | -0.002(0.004) | 0.6880 |  | -0.002(0.004) | 0. 6077 |  | 0.001(0.001) | 0.2030 |
| CSF t-tau | 0.005(0.005) | 0.2838 |  | 0.000(0.000) | 0.7680 |  | -0.001(0.001) | 0.6147 |
| CSF p-tau | 0.000(0.001) | 0.1780 |  | 0.000(0.001) | 0.9782 |  | 0.000(0.002) | 0.5060 |

Data are presented as standardized regression coefﬁcients β and standard error (s.e.) with P values.

Model 1: multiple linear regression model adjusted for age, sex, APOE ԑ4 carrier status, educational level and cognitive diagnosis. Model 2: model 1 with additional inclusion of cardiovascular disease, hypertension, BMI, dyslipidemia and depressive. The interaction was analyzed in model2. Longitudinal analyses interaction term was plasma ApoE×sex×time, cross-sectional analyses was plasma ApoE×sex

**Table 2. The level of CSF and plasma ApoE stratified by APOE genotypes**

|  | *APOE* $\varepsilon$4 non-carriers | | with one *APOE* $\varepsilon$4 allele | | With two *APOE* $\varepsilon$4 allele | P |
| --- | --- | --- | --- | --- | --- | --- |
|  | $\varepsilon2$, $\varepsilon3$ | $\varepsilon3$, $\varepsilon3$ | $\varepsilon2$，$\varepsilon$4 | $\varepsilon3$, $\varepsilon$4 | ε4/ε4 |  |
| CSF | n=26 | n=133 | n=5 | n=109 | n=36 | ＜0.001 |
|  | 9.12±2.36 | 7.14±2.14 | 7.90±2.14 | 6.71±2.23 | 6.40±2.08 |  |
| Plasma | n=28 | n=147 | n=7 | n=130 | n=44 | ＜0.001 |
|  | 80.14±24.90 | 67.59±46.39 | 75.71±31.48 | 48.68±17.46 | 38.98±19.20 |  |

## Supplementary Figures


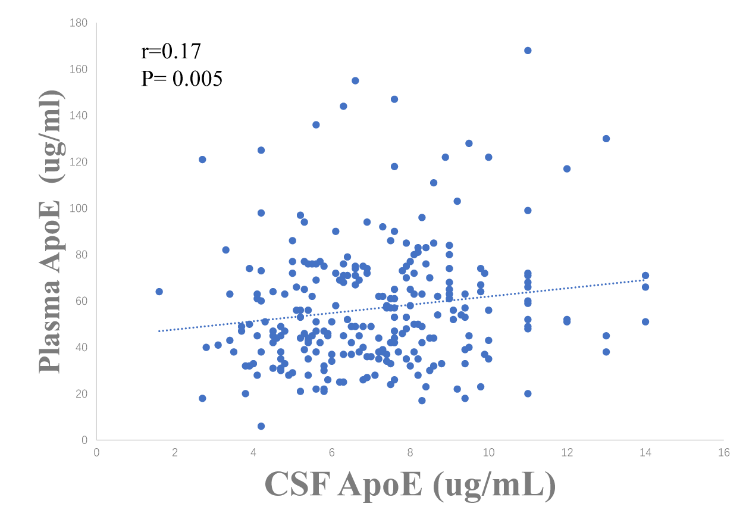


**Supplementary Figure 1.** Association of CSF and plasma ApoE concentration at baseline.

The coefﬁcients r and P values are for Spearman rank correlation in whole cohort.
